# Warnings

**Published:** 1883-09

**Authors:** 


### HALL'S JOURNAL OF HEALTH
AND
### MISCELLANY.

MONTHLY.

E. H. GIBBS, A. M., M. D., EDITOR.

FOUNDED IN 1854.

### WARNINGS.

AN incalculable amount of sickness, suffering and premature deaths would be avoided every year if we could be induced to heed the premonitions which kind nature gives of the coming on of the great enemy, disease. Many a mother has lost a darling child, to her life-long sorrow, by failing to observe the approach of disease in some unusual act or circumstance connected with her offspring.

1. If an adult or child wakes up thirsty in the morning, however apparently well for the moment, or the preceding evening, there will be illness before noon always, infallibly, if not averted by remaining warm in bed, in a cool, well ventilated room, eathing nothing, but drinking plentifully of some hot tea, all day; a little may be eaten in the afternoon by a child. But as long as a person wakes with thirst in the morning there is absence of health—there is fever.

2. If, when not habitual to him, one is waked up early in the morning by an inclination to stool, especially if there is a feeling of debility afterward, it is the premonition of diarrhœa, summer complaint, dysentery or cholera. There should be perfect quietude, etc., as above; in addition, a piece of warm, thick woolen flannel should be wrapped tightly around the abdomen (belly); the drink should be boiled milk; or, far better, eat pieces of ice all the time, and thus keep the thirst perfectly subdued; eat nothing but boiled rice, corn starch, sago or tapioca, and continue all these until the tiredness and thirst are gone, the strength returned, and the bowels have been quiet for twelve hours, returning slowly to the usual activities and diet.

3. If a child is silent, or hangs around its mother to lay its head on her lap, or is unusually fretful, or takes no interest in its former amusement, except for a fitful moment at a time, it is certainly ill, and not slightly so. Send at once for a physician, for you can't tell where or in what form the malady will break out ; and in children especially, you can never tell where any particular ailment will end.

4. When there is little or no appetite for breakfast, the contrary having been the case, the child is ill, and should be put to bed, drinking nothing but warm teas, eating not an atom till noon, then act according to developments.

5. If a child manifests a most unusual heartiness for supper, for several nights in succession, it will certainly be ill, within a week, unless controlled.

6. If there is an instantaneous sensation of sickness at stomach, during a meal, eat not a particle more ; if just before a meal, go out of doors, and keep out in active exercise for several hours, and omit the next meal, for all these things indicate an excess of blood, or bile, and exercise should be taken to work it off, and abstinence, to cut off an additional supply, until the healthful equilibrium is restored.

7. A kind of glimmer before the eyes, making reading or sewing an effort, however well you may feel, will certainly be followed by headache or other discomfort, for there is too much blood, or it is impure; exercise it off in the open air, and omit a meal or two.

8. If you are not called to stool at the accustomed hour (except when traveling, then let things take care of themselves—do nothing), eat not an atom until it is done, for loss of appetite, or nausea, or loose bowels, or biliousness, is certainly impending. Exercise freely out of doors, and drink cold water or hot teas to the fullest desired extent.

9. If there is a most unnatural disposition to exertion, you need rest, quiet, and abstinence ; exercise in weariness never does any good, always harm. But if causelessly despondent, or there is a general feeling of discomfort, the blood is bad ; warm the feet, unload the bowels, eat nothing for twelve hours, and be out of doors all day.

10. If, without any known cause, or special pain, you are exceedingly restless, cannot sleep, or if you do, it is dreamy, disturbed, or distressing, you have eaten too much, or are on the verge of some illness. Take nothing next day but hot drinks and toasted bread, and plenty of out-door exercise. In all these cases, a thorough washing with soap and hot water, and vigorous bodily friction, greatly expedite restoration.

---

Home Carpentry.—"My dear, I can't find the cold chisel, and yet I placed it in this closet myself only yesterday." "Yes, nice place that was to put it such weather as this, and close by the kitchen range, too. Why, it would not stay cold two minutes. It's down cellar in the refrigerator."—*Philadelphia News.*

